# Conducting nationwide cohort COVID-19 serology surveys on a nation with limited resources and decentralized governance: Evidence from Indonesia

**DOI:** 10.1016/j.mex.2024.102609

**Published:** 2024-02-10

**Authors:** Muhammad Noor Farid, Hafizah Jusril, Pandu Riono, Wiji Wahyuningsih, Elmarizha Sekar Utami, Wirabrata, Iwan Ariawan

**Affiliations:** a Department of Biostatistics and Population Studies, Faculty of Public Health, Universitas Indonesia, Depok, Indonesia; b Reconstra Utama Integra, Jakarta, Indonesia; c Centre for Health Resilience and Resource Policy, Ministry of Health, Jakarta, Indonesia

**Keywords:** Seroprevalence, Seroepidemiology, Antibody, Immunology, Vaccination, COVID-19, SARS-CoV-2, Population-based, age and sex-stratified prospective panel study for COVID-19

## Abstract

Knowing the level of SARS-CoV-2 transmission is crucial for decision-making on Coronavirus disease (COVID-19) handling. Guidelines for measuring SARS-CoV-2 antibodies to estimate seroprevalence are conceptually straightforward and internationally available. However, challenges exist for developing countries with limited laboratory capacity, diverse geographical topography, fragmented health information systems and limited resources such as Indonesia. One year after the first case was confirmed in Indonesia, approximately ten infections were undiagnosed or underreported for every reported case. Under those circumstances, we described the method and lessons learned in conducting nationwide cohort COVID-19 serology surveys in a nation with limited resources, such as Indonesia.

• Strong cross-sectoral collaboration between ministries and levels of governance (central and subnational) enables strategic use of civil registration database for efficient sampling.
• All agglomeration districts (highly dense population and urban area) were selected as study sites, and additional non-agglomeration districts were selected using probability-proportional-to-size (PPS).
• Involving the experienced phlebotomist in community health service provider and district laboratory cut down preparation time.

## Graphical abstract

PHO = Provincial Health Office | DHO = District Health Office | Govt lab = Government laboratory, FKM UI = *Fakultas Kesehatan Masyarakat* (Faculty of Public Health) Universitas Indonesia

**Figure:**
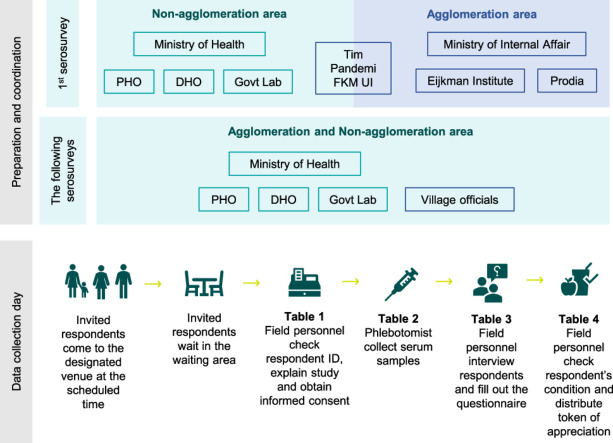

Specifications table

**Table:** 

Subject area:	Medicine and Dentistry
More specific subject area:	Public health, disease surveillance
Name of your method:	Population-based, age and sex-stratified prospective panel study for COVID-19
Name and reference of original method:	Population-based age-stratified seroepidemiological survey for COVID-19 World Health Organization. (2020). Population-based age-stratified seroepidemiological investigation protocol for COVID-19 virus infection, 17 March 2020. World Health Organization. https://apps.who.int/iris/handle/10665/331656. License: CC BY-NC-SA 3.0 IGO
Resource availability:	NA

**Method Details**

## Background

Indonesia is the world's largest archipelago, where 276.4 million populations reside across 34 provinces and 514 districts. By December 2022, Indonesia had experienced at least three waves, resulting in 6.7 million confirmed COVID-19 cases [1]. Like other developing countries, the COVID-19 pandemic reveals Indonesia's frail health system, resulting in more than 60,000 new cases and 2,000 deaths per day in one period of time [2]. Indonesia witnessed substandard contact tracing, delayed testing-laboratory-reporting for case confirmation, and poor social distancing and isolation compliance. Indeed, handling the COVID-19 pandemic demands the utmost priority in health, economics, and politics. Correspondingly, numerous advocacy and measures are in place to transform then strengthen the overall health system. Indonesia's COVID-19 response has progressed considerably, and the Government of Indonesia have successfully administered 203 million the 1st vaccine dose by December 2022 [3]. One of the notable measures was the use of national serosurveys for vaccination and other policies, serving as a living manifestation of evidence-based policy

Indonesia's most extensive national cohort survey was initially a one-time cross-sectional investigation in December 2021. Continuous advocacy measures succeeded in having the Ministry of Health adopts and leads the following serosurveys visiting the same respondents. Overall, the two primary objectives of the serosurveys are to (1) estimate the proportion of people with SARS-CoV-2 antibodies by age group, sex, ever-diagnosed with COVID-19 and vaccination status; and (2) approximate distribution of antibody titre by ever diagnosed with COVID-19 and vaccination status. The secondary objectives include assessing the changes in the proportion of people with SARS-CoV-2 antibodies and antibody titre.

This article details the conceptual and technical serosurvey as inspired by the World Health Organization (WHO) serosurvey guideline. The WHO's direction on the population-based age-stratified seroepidemiological investigation on COVID-19 [4] is straightforward, with options to facilitate varied settings, resources and capacities. However, Indonesia is challenged by its widely diverse geographical topography, varied health system capacity, and dimensions of health inequality [5]. Furthermore, Indonesia adopts a decentralized government system, placing shared responsibilities between central, provincial, and district health offices on planning, and managing service delivery. However, during outbreaks, this arrangement changes. Indonesian law and regulation mandates the head of the respective area (governor, major, regent) to lead the outbreak handling. In other words, when an outbreak occurs, the control and management of communicable diseases are no longer solely led by the health sector; creating a large-scale coordination. In this method article, we outline the technical steps for conducting a population-based, age and sex-stratified national-level serosurvey in Indonesia, along with the lessons learned applicable to other developing countries.

## Study design and setting

The nationwide serosurvey adopts a prospective panel study design with 6-months intervals. The Ministry of Health, in collaboration with the Ministry of Internal Affairs, Eijkman Institute for Molecular Biology, and Prodia conducted the first serosurvey in December 2021. By this time, the vaccination program has been rolled out for six months to the general population, specifically since June 2021. The Ministry of Health leads subsequentserosurveys in June 2022 and January 2023. The independent team, namely *Tim Pandemi FKM UI*, lead the sampling method and data analysis. Data is collected within one to two weeks in each district, depending on geographical topography and district capacities.

**Fig. 1:**
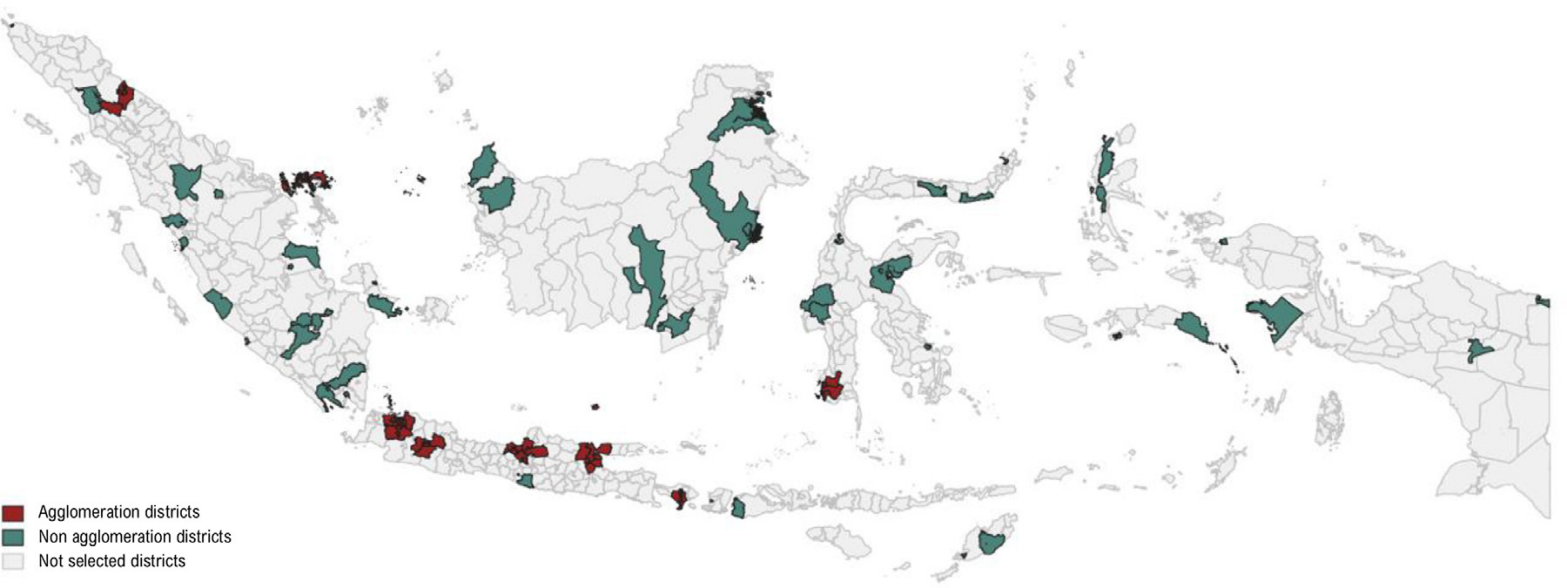
The selected 100 districts as Indonesia's national serosurvey sites.

Serosurvey is conducted on 100 selected districts located across Indonesia, all 34 provinces. Ministry of Internal Affairs lists 47 districts as agglomeration areas – all included as serosurvey sites. The non-agglomeration districts are selected using probability-proportional-to-size (PPS) sampling from all non-agglomeration districts with the population as size, considering urban and rural characteristics. This resulted in 53 selected non-agglomeration districts as serosurvey sites (Fig. 1).

## Participants

All individuals aged one year and older officially registered as Indonesian citizens (have citizen ID) are the population of this national serosurveys. We exclude the selected personnel who refuse, do not consent to participate, or do not present on the data collection sites. Individuals with contraindications to venepuncture or who are hospitalized are withdrawn from studies. Next, the survey excluded those who pass away or migrate to other cities on the following surveys. Finally, those who did not participate in the prior serosurvey are dropped on the following surveys.

## Sample size

The first serosurvey aimed to collect 10,280 respondents in the agglomeration area and 11,600 respondents in the non-agglomeration area (Table 1). The Government of Indonesia broadly defines an agglomeration area as an area where population mobility and economic activity are concentrated.The minimum sample size was calculated based on the expected prevalence of anti-SARS CoV-2 antibodies in each area using the following formula:

$$n=\frac{NZ^{2}p\left(1-p\right)}{d^{2}\left(N-1\right)+Z^{2}p\left(1-p\right)}\times \frac{deff}{rr}$$

$$p=\frac{c}{N}\times \frac{1}{rep}d=rp\times p$$

with, *n* = sample size, Deff = design effect, *N* = population size, *p* = estimated proportion, *q* = 1-p, *d* = desired precision or absolute level of precision, rr = response rate, rep = reporting rate. Assumptions applied are 8.5% national seroprevalence, absolute precision of 0.85% (relative precision of 10%), a design effect of 2, response rate of 80%, and 95% confidence interval. All serosurvey respondents are revisited in the following serosurveys. In each phase, we expect up to 20% non-response because of population migration, mortality, morbidity, and scheduling conflict.

**Table 1:** Number of samples.

Sampling unit	Agglomeration area	Non-Agglomeration area	Total samples
District	47	53	100
Village	514	580	1,094
Sample/ respondent	10,280	11,600	21,880

The 1st serosurvey was conducted in December 2021 and collected 20,501 (93.7%) samples. The following serosurvey was conducted six months later, specifically in July 2022 and collected 17,315 (84.5%) samples. The latest nationwide serosurvey on January 2023 analyzed 16,286 samples, reaching 94% target.

### Sampling

Using a probability approach, we adopt a stratified two-stage cluster sampling design in each selected district. First, villages are selected by PPS sampling with the number of households as size. The sampling frame uses the previous village census or *Pendataan Potensi Desa* (PODES) [6] conducted by BPS-Statistics Indonesia/*Badan Pusat Statistik* (BPS) in 2018. Indonesian villages have diverse areas, geographical topography, and local health system capacity. For this reason, the number of villages is calculated upon the pre-determined 20 respondents for each village. The number of twenty respondents is determined upon feasibility, capacity and quality of survey estimation. The calculation results in total 514 selected villages in agglomeration districts and 580 selected villages in non-agglomeration districts. Second, the twenty individuals of the primary sample in each village are selected using systematic random sampling stratified by sex and age groups. The individual-sample selection use lists by the Civil Registry Office, one of the district-level representatives of the Ministry of Internal Affairs.

Prior to data collection, local healthcare provider staff collaborate with the village staff, community health workers, and cadres to cross-check the sample list and identify the sample contact. On the first serosurvey, additional 60 individuals are generated as replacement lists, stratified by sex and age groups (1–14 years, 15–49 years, and >50 years). If any of the initially selected samples cannot be found, they will be replaced with individuals from the backup list. The contact list obtained during the first serosurvey serves as a reference point for subsequent serosurveys.

### Data collection personnel

All field personnel are experienced health staff and professional phlebotomists. On the 1st serosurvey (December 2021), district health offices led data collection and logistics management in the non-agglomeration area, whereas the office of internal affairs and Prodia (private laboratory) led data collection in the agglomeration area. On the following serosurveys, district health offices and government laboratories lead the data collection and sample testing on all sites. Village staff, in collaboration with local healthcare provider staff and community health service staff, send the formal invitation to the selected samples. On the D-day, different field personnel are assigned for registration and ID confirmation, serum samples collection, enumerator for interview, and overall documentation on each data collection site.

### Data collection procedure

In the first serosurvey, two data collection procedures were employed due to varied infrastructure circumstances. Where the laboratory is available, the laboratory district invites the selected respondents to the venue. In other districts, respondents are invited to the assigned venue, and the specimen is transported to the laboratories. Venues include village hall and community health centre. The head of village or primary health service provider staff sends the invitation before the D-day. Respondents with a clashing schedule will be arranged for data collection on the remaining dates at the other venue within a district. Respondents are interviewed using paper-based questionnaires, which the enumerator enters into designated software.

**Fig. 2:**
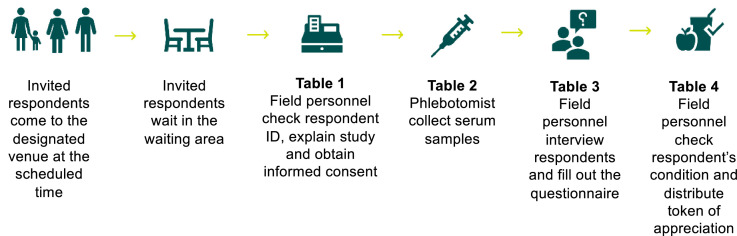
Data collection procedure.

On the-D day, the respondents are requested to bring their ID to the venue to verify the respondent's identity. In each venue, several tables were set in order. First, field personnel confirm the respondents' identity, explain the study and obtain written consent from the respondents or his/her guardian. Respondents then are directed to the specimen collection table, where approximately 3–6 millilitres of blood are drawn by venepuncture. Finally, respondents are interviewed by an enumerator at separate tables (Fig. 2).

We use a web-based application for both questionnaire and laboratories data entries. The application provides initial data checking by using skip logic. Depending on the network strength and circumstances on the venue, the enumerator could entry data on the data collection venue or other designated venue on the d-day of data collection.

### Serum and plasma handling

After blood is drawn, the collected venous blood samples are stored in a serum separator tube (SST). In each district, serums are separated by phlebotomist on the d-day of data collection upon several scenarios. Where a portable centrifuge is available on data collection sites, serum samples are centrifuged on-site at 2,000 rpm for 10 min directly after data collection. In other circumstances, serum samples are transported in wet ice cool box (at 2–4 °C) to the district laboratory, where the serum is separated on the same day. Each respondent's sample was stored into two cryotubes. One cryotube receives 500 ul of serum and is labelled with a sticker, while the remaining serum is placed into another cryotube. Samples then are stored in a freezer at -20 °C or lower (at -80 °C) at the district laboratory.

Once data collection in one district is concluded, serum samples are transported to the central laboratory, *Prof. Dr. Sri Oemijati* Network and Infectious Disease Laboratory, in Jakarta. The serum specimens are sent in a cooled state at a temperature of 2–4 °C, utilizing a cool box and ice pack gel, in accordance with WHO guidelines.

### Characteristics and interpretation of serologic assays

Test use Elecsys Anti-SARS-CoV-2 S (Roche Diagnostics International Ltd, Rotkreuz, Switzerland) [7]- the immunoassay that detects IgG antibodies against the SARS-CoV-2 nucleocapsid protein and binding domain (RBD-receptor binding domain) of the SARS-CoV-2 spike protein. On the first serosurvey, an automated Cobas e601 immunoassay analyzer (Roche Diagnostics International Ltd, Rotkreuz, Switzerland, limit of detection) [8] test serum samples from the agglomeration area. Serum samples from non-agglomeration area are tested using an automated Cobas e411 immunoassay analyzer (Roche Diagnostics International Ltd, Rotkreuz, Switzerland [9]. The following serosurveys continue to use Cobas e411 immunoassay analyzer. Both have a limit of detection of 0.40 U/mL.

Serum testing is based on Electro-Chemiluminescence Immunoassay (ECLIA) technique. Previous studies report the ECLIA test for SARS-CoV-2 antibodies reaches at least 89.5% sensitivity and 99.1% specificity [10], [11], [12]. Calibration and internal quality control are performed according to the manufacturer's recommendations. We retest each 10 % of positive and negative serum samples using the same assay for quality control.

## Variables

Both serum samples and epidemiological data are collected from each respondent. The interviews collect sociodemographic characteristics, places visited in the past week, history of symptoms in the past month, records of contact with a confirmed COVID-19 case or suspect in the past month, ever-diagnosed COVID-19, history of COVID-19 vaccination, comorbidities, risk factors and preventive behaviour of COVID-19. We identified notable recall bias on vaccination history. Likewise, coverage bias on ever-diagnosed COVID-19 question is expected due to its asymptomatic characteristics and COVID-19 shame. Following that, the national database on COVID-19 vaccination and confirmed COVID-19 cases are analyzed and linked to the serosurvey respondents using citizen ID.

## Statistical analysis

Serum sample data and questionnaire data are matched using a barcode. The prevalence of population with SARS-CoV-2 antibody and average antibody titre was estimated with 95% confidence interval, taking weighted clustering stratification in the calculation. The prevalence distribution is assessed across region, district, sex and age groups. Antibodies triter are estimated across age groups, sex, region and vaccination histories. All estimates are sex and age group weighted to account for higher non-response rates in males and younger age groups. Regression is performed to control confounders. All analyses are performed and visualised using Stata 17.

## Ethics statements

The Ethics Committee approved each phase of the cohort serosurvey. The Eijkman Institute for Molecular Biology and Health Development Policy Agency Ethical Committee approved the first phase of the survey (December 2021). The second survey (June 2022) received ethical clearance from the Health Development Policy Agency Ethical Committee. The third survey, conducted on January 2023, is submitted to Universitas Atmajaya Ethic Board. Written consent is obtained prior to data collection from individuals aged 18 years or older and caregiver of individuals between 1 and 17 years.

## CRediT authorship contribution statement

**Muhammad Noor Farid:** Conceptualization, Methodology, Software, Formal analysis, Supervision. **Hafizah Jusril:** Validation, Data curation, Writing – original draft, Visualization. **Pandu Riono:** Conceptualization, Methodology, Validation, Investigation, Funding acquisition. **Wiji Wahyuningsih:** Data curation, Software, Visualization, Project administration. **Elmarizha Sekar Utami:** Data curation, Software, Visualization, Project administration. **Wirabrata:** Resources. **Iwan Ariawan:** Conceptualization, Methodology, Investigation, Funding acquisition, Formal analysis, Supervision.

## Declaration of competing interest

The authors declare the following financial interests/personal relationships which may be considered as potential competing interests: One of the authors is affiliated with the funding organization. While the development of the manuscript did not entail direct involvement from the funders during its preparation, the publication of the manuscript is subject to approval by the funding organization.

## Data Availability

No data was used for the research described in the article.

## References

[1] Satuan Tugas Penanganan COVID-19. Peta sebaran. 2022. https://covid19.go.id/peta-sebaran#.2022).

[2] Satuan Tugas Penanganan COVID-19. Perkembangan kasus terkonfirmasi positif Covid-19 per-hari nasional. 2022. https://covid19.go.id/peta-sebaran#.

[3] Minisrty of Health Republic of Indonesia. Vaksinasi COVID-19 Nasional 2022. https://vaksin.kemkes.go.id/#/vaccines2022).

[4] World Health Organization (2020).

[5] World Health Organization (2017).

[6] Badan PusatStatistik (2018).

[7] Roche Diagnostics International. Elecsys® Anti-SARS-CoV-2 S. 2022. https://diagnostics.roche.com/global/en/products/params/elecsys-anti-sars-cov-2-s.html#relatedProducts.

[8] Roche Diagnostics International. Cobas e 601 module for immunoassay tests. 2022. https://diagnostics.roche.com/global/en/products/instruments/cobas-e-601-ins-461.html#productInfo.

[9] Roche Diagnostics International. Cobas e 411 analyzer for immunoassay tests (disk system). 2022. https://diagnostics.roche.com/global/en/products/instruments/cobas-e-411-ins-502.html2022).

[10] Afzal N., Tariq N., Raza S., Shakeel D. (2021). Diagnostic accuracy of electro-chemiluminescence immunoassay anti-SARS-CoV-2 serological test. Cureus.

[11] Kolesova O., Tomassetti F., Cerini P. (2022). Evaluation of ECLIA antigen detection tests as screening methods for COVID-19 in comparison with molecular analysis. Irish J. Med. Sci..

[12] Weber M.C., Risch M., Thiel S.L. (2021). Characteristics of Three Different Chemiluminescence Assays for Testing for SARS-CoV-2 Antibodies. Dis. Markers.

